# Combination of thrombin-antithrombin complex, plasminogen activator inhibitor-1, and protein C activity for early identification of severe coagulopathy in initial phase of sepsis: a prospective observational study

**DOI:** 10.1186/cc13190

**Published:** 2014-01-13

**Authors:** Kansuke Koyama, Seiji Madoiwa, Shin Nunomiya, Toshitaka Koinuma, Masahiko Wada, Asuka Sakata, Tsukasa Ohmori, Jun Mimuro, Yoichi Sakata

**Affiliations:** 1 Division of Intensive Care, Department of Anesthesiology & Intensive Care Medicine, Jichi Medical University School of Medicine, 3311-1 Yakushiji, Shimotsuke, Tochigi 329-0498, Japan; 2 Research Divisions of Cell and Molecular Medicine, Center of Molecular Medicine, Jichi Medical University School of Medicine, 3311-1 Yakushiji, Shimotsuke, Tochigi 329-0498, Japan

## Abstract

**Introduction:**

Current criteria for early diagnosis of coagulopathy in sepsis are limited. We postulated that coagulopathy is already complicated with sepsis in the initial phase, and severe coagulopathy or disseminated intravascular coagulation (DIC) becomes overt after progressive consumption of platelet and coagulation factors. To determine early diagnostic markers for severe coagulopathy, we evaluated plasma biomarkers for association with subsequent development of overt DIC in patients with sepsis.

**Methods:**

A single-center, prospective observational study was conducted in an adult ICU at a university hospital. Plasma samples were obtained from patients with sepsis at ICU admission. Fourteen biomarkers including global markers (platelet count, prothrombin time, activated partial thromboplastin time, fibrinogen and fibrin degradation product (FDP)); markers of thrombin generation (thrombin-antithrombin complex (TAT) and soluble fibrin); markers of anticoagulants (protein C (PC) and antithrombin); markers of fibrinolysis (plasminogen, α_2_-plasmin inhibitor (PI), plasmin-α_2_-PI complex, and plasminogen activator inhibitor (PAI)-1); and a marker of endothelial activation (soluble E-selectin) were assayed. Patients who had overt DIC at baseline were excluded, and the remaining patients were followed for development of overt DIC in 5 days, and for mortality in 28 days.

**Results:**

A total of 77 patients were enrolled, and 37 developed overt DIC within the following 5 days. Most patients demonstrated hemostatic abnormalities at baseline with 98.7% TAT, 97.4% FDP and 88.3% PC. Most hemostatic biomarkers at baseline were significantly associated with subsequent development of overt DIC. Notably, TAT, PAI-1 and PC discriminated well between patients with and without developing overt DIC (area under the receiver operating characteristic curve (AUROC), 0.77 (95% confidence interval, 0.64 to 0.86); 0.87 (0.78 to 0.92); 0.85 (0.76 to 0.91), respectively), and using the three together, significantly improved the AUROC up to 0.95 (vs. TAT, PAI-1, and PC). Among the significant diagnostic markers for overt DIC, TAT and PAI-1 were also good predictors of 28-day mortality (AUROC, 0.77 and 0.81, respectively).

**Conclusions:**

Severe coagulation and fibrinolytic abnormalities on ICU admission were associated with subsequent development of overt DIC. A single measurement of TAT, PAI-1, and PC activity could identify patients with ongoing severe coagulopathy, early in the course of sepsis.

## Introduction

Sepsis is frequently complicated with coagulopathy [1]. The severity of sepsis-associated coagulopathy is variable, ranging from subclinical abnormalities that are detectable by a mild decrease in platelet count and prolongation of global clotting times, to severe forms of coagulopathy or disseminated intravascular coagulation (DIC) [2]. The incidence of DIC is up to 25 to 50% in patients with sepsis [3].

Septic DIC is characterized by systemic intravascular activation of coagulation, and microvascular endothelial injury with impaired anticoagulation and insufficient fibrinolysis, which leads to widespread thrombosis in microvasculature. In sepsis, DIC has a feature of vascular endothelial dysfunction, as well as being an etiological factor in the failure of other organs: excessive thrombin generation and subsequent fibrin deposition exacerbate inflammation and ischemia, contributing to organ damage [4]. A number of studies have reported that DIC is an independent risk factor for organ dysfunction and mortality in patients with sepsis [2,3,5]. DIC might, therefore, be an important therapeutic target in the management of sepsis, and the development of reliable methods for early identification of DIC is a high priority.

However, the early diagnosis of sepsis-associated coagulopathy and evaluation of its severity is still challenging [6]. Currently, the overt DIC criteria of the International Society on Thrombosis and Haemostasis (ISTH) are the diagnostic standard for severe coagulopathy in sepsis [4]. Although the ISTH criteria for overt DIC are simple and widely used, and shown to be associated with organ failure and mortality, they have limited application in the early phase of sepsis to improve outcome [7,8]. The ISTH overt DIC criteria use global markers, such as prothrombin time (PT) and platelet count for scoring. The coagulation factors and platelets are consumed and decrease over time because of progressive thrombin generation and endothelial injury, thus it takes several days to reveal their abnormalities and fulfill the overt DIC criteria in the course of sepsis [9,10]. Furthermore, introduction of the concept of pre-DIC, which is considered as the stage prior to overt DIC, has failed to predict disease progression. An ISTH subcommittee defined non-overt DIC as compensated coagulopathy, or pre-stage DIC, for the early diagnosis of overt DIC [4]. However, previous studies have shown that only 10 to 30% of patients with non-overt DIC progressed to overt DIC, although the mortality rates were similar between patients with non-overt and overt DIC [6,11].

In the past decade, there has been increasing evidence that inflammation and coagulation play pivotal roles in the pathogenesis of sepsis [12,13]. Pro-inflammatory cytokines produced by the host response against infection stimulate tissue factor expression and lead to activation of coagulation. An activated coagulation system in turn modulates inflammatory activity through specific receptors, such as protease-activated receptors. Considering that excessive crosstalk between inflammation and coagulation is ongoing from the onset of sepsis, severe coagulopathy may have developed early in the course.

The objective of this study was to identify hemostatic biomarkers that can be used for early diagnosis of severe coagulopathy in patients with sepsis. We postulate that severe coagulopathy has already developed in the initial phase of sepsis, and is related to the subsequent fulfillment of the criteria for overt DIC [14]. We, therefore, evaluated the association between plasma biomarkers measured at the time of intensive care unit (ICU) admission and development of overt DIC in the following five days. We also investigated the hemostatic biomarkers as predictors for 28-day mortality.

## Material and methods

### Study design and setting

This was a single-center, prospective observational study, that was conducted in a 12-bed medicosurgical ICU at a university hospital from January 2012 to June 2013. The study was approved by the Institutional Research Ethics Committee of Jichi Medical University, and informed consent was obtained from the patients or their families.

The consecutive patients who were admitted to the ICU because of sepsis, and without overt DIC on ISTH criteria at the time of ICU admission, were enrolled. Sepsis was defined according to the 2001 International Sepsis Definitions Conference [15]. Exclusion criteria were: age <18 years, presence of decompensated cirrhosis (Child-Pugh class B or C), hematological disorders, chronic renal failure on hemodialysis, and history of therapeutic anticoagulation or blood transfusion during the preceding four weeks.

Clinical and demographic data, including age, sex, comorbidity and Acute Physiology and Chronic Health Evaluation (APACHE) II scores [16], were recorded on ICU admission. Sequential Organ Failure Assessment (SOFA) score [17] except for coagulation (platelet count), and overt DIC score on ISTH criteria were determined daily. ISTH non-overt DIC score, and acute DIC score established by the Japanese Association for Acute Medicine (JAAM) [18] were also calculated daily as early diagnostic systems for DIC.

The primary endpoint was the development of overt DIC within the first five days of ICU stay. A score ≥5 on the ISTH criteria was defined as overt DIC. The secondary endpoint was 28-day all-cause mortality. Plasma samples were drawn from the eligible patients within 6 h of ICU admission, and the patients were followed for 5 days for overt DIC score and 28 days for mortality.

### Biomarker measurements

Plasma biomarkers were measured at the time of ICU admission (Day 0) as baseline, and on days 1 to 3. We classified 14 biomarkers into five categories: global markers (platelet count, prothrombin time (PT) and PT-international normalized ratio (PT-INR), activated partial thromboplastin time, fibrinogen, fibrin degradation product (FDP)); markers of thrombin generation (thrombin–antithrombin complex (TAT), soluble fibrin (SF)); markers of anticoagulants activity (protein C (PC), antithrombin (AT)); markers of fibrinolytic activity (plasminogen, α_2_-plasmin inhibitor (PI), plasminogen activator inhibitor (PAI)-1, plasmin–α_2_-PI complex (PIC)); and a marker of endothelial activation (soluble E-selectin (sES)).

Blood samples were collected heparin-free and centrifuged at 2,500 rpm at 4°C in citrated tubes. Global markers, TAT, PC, AT, plasminogen, α_2_-PI and PIC were assayed using the CS-2100i automatic coagulation analyzer (Sysmex, Hyogo, Japan) immediately after the samples were collected. Berichrom assays (Siemens Healthcare Diagnostics, Tokyo, Japan) were used for PC, AT, plasminogen and α2-PI activities, and TAT/PIC test F enzyme immunoassay (Sysmex) were used for measurements of TAT and PIC levels, respectively. SF, PAI-1 and E-selectin were measured with the stored samples, which were frozen at -80°C within 2 h of collection, using iatroSF, tPAI test and sES latex photometric immunoassay, respectively (Mitsubishi Chemical Medience, Tokyo, Japan).

### Patient management

Our facility provides 24-h coverage by attending ICU physicians. Management of patients followed the Surviving Sepsis Campaign Guidelines (SSCG) with the goal of initial resuscitation and infection control [19]. Patients received mechanical prophylactic treatment without concomitant low-dose heparin, until no active bleeding or severe coagulopathy was confirmed. Antithrombin substitution therapy was at the discretion of the ICU physicians, limited for the patients with AT activity <50% after the plasma samples at baseline were collected. The patients with bleeding risk or complications were transfused with platelet concentrate or fresh frozen plasma as decided by the ICU physicians.

### Data analysis

The study population was grouped according to the development of overt DIC. Statistical differences between the groups were analyzed using Wilcoxon rank-sum test for non-normally distributed variables, and the χ^2^, or Fisher’s exact test for categorical variables as appropriate. Biomarker abnormalities were defined as values higher than the upper limit of normal, or lower than the lower limit of normal, which were used in practice at our institution. Receiver operating characteristic (ROC) curve analysis was performed to calculate the area under the receiver operating characteristic curve (AUROC) of the 14 biomarkers at baseline for the development of overt DIC, and of those at baseline and at Day 2 for 28-day mortality. The AUROC for APACHE II score and pre-DIC scores (by ISTH non-overt DIC, and JAAM acute DIC criteria) at baseline were also calculated for comparison. The best cutoff values were calculated to maximize the sum of sensitivity and specificity. Positive predictive value (PPV) and negative predictive value (NPV) were also calculated. To assess the bivariable association among biomarkers, Spearman rank correlation coefficients (*r* value) along with the associated *P-*value were calculated, and *r* <0.5 was considered as no evidence of collinearity. A multivariate logistic regression model based on a forward stepwise method was used to identify the best combination to discriminate the development of overt DIC. To assess the impact of biomarkers on survival, Kaplan-Meier estimates were used to illustrate trends in 28-day mortality and the log-rank test was performed. All *P-*values were two-tailed, and *P* <0.05 was considered statistically significant. Data were analyzed using JMP version 10 (SAS Institute, Tokyo, Japan).

## Results

### Patient characteristics and outcomes

One hundred, eleven patients were admitted to the ICU because of sepsis during the study period. Thirty-four patients were excluded according to the study criteria, and the remaining 77 patients were enrolled. The baseline characteristics and prognosis of the study population are described in Table 1. Of 77 patients with sepsis, 37 (48.1%) developed overt DIC within five days of their ICU stay. Patients who newly developed overt DIC were more severely ill with a higher APACHE II score, maximum SOFA scores and 28-day mortality, compared with patients who did not develop overt DIC. No therapeutic heparin was administered during the study period. Prophylactic low-dose heparin was used more frequently in patients without DIC than in those who developed overt DIC (50.0 vs. 10.8%, *P* = 0.0001). Platelet concentrate, fresh frozen plasma and antithrombin were more frequently administered to patients who developed overt DIC than to those who did not (24.3 vs. 0%, *P* = 0.0001; 29.7 vs. 7.5%, *P* = 0.012; 56.8 vs. 5.0%, *P* <0.0001, respectively).

**Table 1:** Baseline characteristics and outcomes of the 77 patients with sepsis

	**All patients (n = 77)**	**Develop DIC (n = 37)**	**No DIC (n = 40)**	***P-*** value*
**Demographics**				
Age (years)	69.9 ± 12.9	70.7 ± 13.2	69.1 ± 12.7	0.58
Male	42 (54.5)	16 (43.2)	26 (65.0)	0.069
**Source of sepsis**				
Pulmonary infection	15 (19.5)	7 (18.9)	8 (20.0)	0.91
Abdominal infection	43 (55.8)	22 (59.5)	21 (52.5)	0.54
Urinary tract infection	5 (6.5)	3 (8.1)	2 (5.0)	0.58
Soft tissue infection	11 (14.3)	3 (8.1)	8 (20.0)	0.13
Blood stream infection	2 (2.6)	2 (5.4)	0 (0.0)	0.084
**Comorbidities**				
IHD	7 (9.1)	2 (5.4)	5 (12.5)	0.27
CHF	2 (2.6)	0 (0.0)	2 (5.0)	0.11
Arrhythmia	3 (3.9)	3 (8.1)	0 (0.0)	0.033
COPD	6 (7.8)	1 (2.7)	5 (12.5)	0.094
CKD	10 (13.0)	6 (16.2)	4 (10.0)	0.42
CVD	3 (3.9)	2 (5.4)	1 (2.5)	0.51
**Severity of illness**				
APACHE II score	25.4 ± 7.9	28.8 ± 8.2	22.2 ± 6.1	0.0002
**Organ dysfunction (days 0 to 5)**				
max SOFA score**	9 (7 to 11)	10 (9 to 14)	7 (4 to 9)	0.0001
**Prognosis**				
ICU-free days	18 (10 to 21)	16 (0 to 19)	21 (17 to 23)	0.0001
28-day mortality	15 (19.5)	13 (35.1)	2 (5.0)	0.0005

Data are expressed as mean ± SD, median (interquartile range), or No. (%).

APACHE, acute physiology and chronic health evaluation; CHF, chronic heart failure; CKD, chronic kidney disease; COPD, chronic obstructive pulmonary disease; CVD, cerebrovascular disease; IHD, ischemic heart disease.

*Comparison of groups with and without subsequent development of overt DIC.

**maximum SOFA scores except for score of coagulation (platelet count) during the first five days of ICU stay.

### Evidence of biomarker abnormalities at baseline and subsequent changes over time

The majority of the 77 patients with sepsis presented with plasma biomarker abnormalities at the time of admission (Day 0), as indicated by elevated TAT (98.7% of patients) and FDP (97.4% of patients), and decreased activity of PC (88.3% of patients, Table 2). In contrast, decreased platelet count, prolonged PT-INR or decreased level of fibrinogen was not frequently observed on Day 0 in patients with sepsis.

**Table 2:** Plasma biomarkers at baseline (Day 0) in patients with sepsis

	**Normal range**	**All patients**		**Develop DIC**	**No DIC**	
		**Median level**	**Abnormal patients (%)**	**Median level**	**Median level**	***P-*** value*
**Global markers**						
Platelet (× 10^3^/μL)	130 to 369	163 (118 to 205)	33.8^a^	140 (108 to 184)	176 (136 to 228)	0.036
PT-INR	0.9 to 1.2	1.25 (1.15 to 1.37)	55.8^b^	1.29 (1.17 to 1.38)	1.21 (1.13 to 1.31)	0.091
APTT (sec)	23.1 to 36.3	39.5 (32.2 to 48.9)	62.3^b^	42.7 (35.4 to 49.5)	37.7 (31.3 to 42.7)	0.096
Fibrinogen (mg/dL)	129 to 371	395 (249 to 544)	25.9^a^	299 (225 to 481)	419 (319 to 565)	0.041
FDP (μg/mL)	0 to 5.0	16.7 (10.4 to 28.9)	97.4^b^	20.5 (11.7 to 44.1)	15.6 (8.4 to 22.1)	0.011
**Thrombin generation**						
TAT (ng/mL)	<2.4	12.5 (7.2 to 20.1)	98.7^b^	19.5 (10.5 to 25.8)	8.4 (5.7 to 12.9)	<0.0001
SF (μg/mL)	<7.0	10.5 (5.3 to 24.2)	66.2^b^	13.9 (7.9 to 29.3)	7.4 (3.2 to 17.3)	0.013
**Anticoagulant activity**						
PC (%)	67 to 129	46.2 (34.1 to 59.5)	88.3^a^	36.6 (28.1 to 44.9)	59.1 (46.7 to 65.6)	<0.0001
AT (%)	75 to 125	51.8 (38.4 to 63.2)	88.3^a^	42.8 (31.3 to 54.8)	58.2 (48.1 to 72.5)	0.0001
**Fibrinolytic activity**						
Plasminogen (%)	85 to 120	60.2 (43.6 to 73.7)	85.7^a^	48.1 (30.3 to 66.1)	67.0 (57.1 to 84.6)	0.0001
α2-PI (%)	83 to 115	67.3 (52.1 to 82.8)	74.1^a^	54.1 (40.5 to 67.6)	78.6 (67.1 to 88.7)	<0.0001
PAI-1 (ng/mL)	<50.0	154.7 (60.7 to 533.1)	81.8^b^	531.6 (191.1 to 992.6)	77.6 (40.8 to 154.7)	<0.0001
PIC (μg/mL)	<0.9	1.0 (0.7 to 1.8)	54.5^b^	1.0 (0.6 to 2.7)	1.1 (0.8 to 1.5)	0.99
**Endothelial activation**						
sES (ng/mL)	<29.7	55.2 (35.9 to 101.1)	83.1^b^	65.3 (34.8 to 144.8)	49.5 (36.9 to 72.9)	0.17

α2-PI, α2-plasmin inhibitor activity; APTT, activated partial thromboplastin time; AT, antithrombin activity; DIC, disseminated intravascular coagulation; FDP, fibrin degradation products; PAI-1, plasminogen activator inhibitor-1; PC, protein C activity; PIC, plasmin-α2-plasmin inhibitor complex; PT-INR, prothrombin time-international normalized ratio; sES, soluble E-selectin; SF, soluble fibrin; TAT, thrombin-antithrombin complex.

*Comparison of groups with and without subsequent development of overt DIC. ^a^Percentage of patients with values lower than the lower limit of normal. ^b^Percentage of patients with values higher than the upper limit of normal.

**Figure 1:**
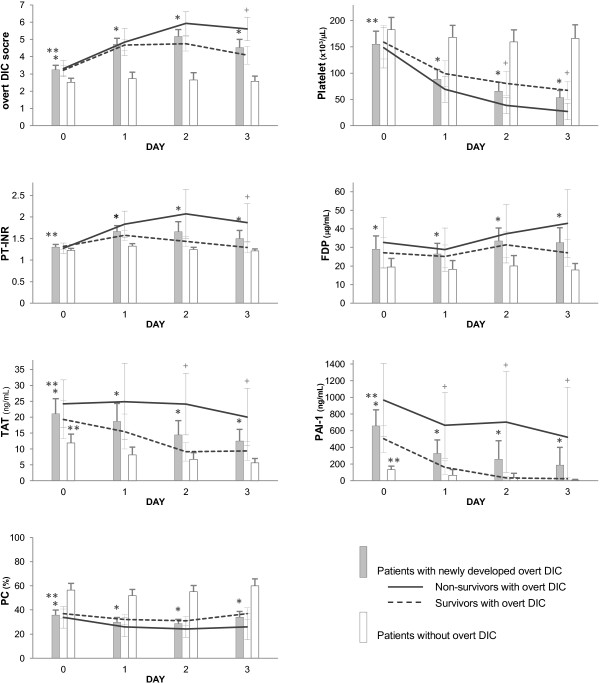
**Time course of overt DIC scores and hemostatic biomarkers from baseline to Day 3.** Overt disseminated intravascular coagulation (DIC) scores, platelet count, prothrombin time-international normalized ratio (PT-INR), fibrin degradation product (FDP), thrombin-antithrombin complex (TAT), plasminogen activator inhibitor-1 (PAI-1) and protein C (PC) for patients with and without subsequent development of overt DIC (gray vs. white bars), and for survivors (dotted line) and non-survivors (solid line) among patients with overt DIC. Data are expressed as mean and 95% CI. *P <0.05 between patients with and without overt DIC on the same day. **P <0.05 between patients on Day 0 versus Day 2. ^+^*P* <0.05 between survivors and non-survivors with overt DIC on the same day.

Plasma biomarkers of platelet, PT-INR, FDP, TAT, PAI-1 and PC over time (days 0 to 3) in patients with and without subsequent development of overt DIC are shown in Figure 1 (other studied biomarkers are shown in Additional file 1). On Day 0, there were marked increases in TAT and PAI-1, and decreases in PC, plasminogen and α_2_-PI activities, which were particularly marked in patients with subsequent development of overt DIC. Notably, TAT and PAI-1 were the highest on Day 0 and gradually returned to normal in patients who developed overt DIC (TAT on Day 0 vs. Day 2, *P* = 0.013; PAI-1, *P* = 0.0035), whereas platelet count and PT-INR were around the normal range on Day 0 and exacerbated until days 2 to 3 (platelet on Day 0 vs. Day 2, *P* <0.0001; PT-INR, *P* = 0.0043).

### Discrimination capacity of plasma biomarkers at baseline for subsequent development of overt DIC

We conducted ROC curve analysis to evaluate the ability of biomarkers to discriminate among patients who subsequently developed overt DIC and those who did not. The AUROCs and best calculated cutoff values, PPV and NPV, are shown in Table 3. The AUROCs and PPVs for the development of overt DIC were high for TAT, PC, AT, plasminogen, α2-PI and PAI-1. For the comparison between discrimination abilities of plasma biomarkers and those of severity of illness, and pre-DIC scores at baseline, the AUROCs and PPVs were also calculated for APACHE II scores (AUROC, 0.72, (95% confidence interval, 0.61 to 0.82); PPV, 0.62), ISTH non-overt DIC scores (AUROC, 0.71 (0.59 to 0.80); PPV, 0.58), and JAAM DIC scores (AUROC, 0.68 (0.55 to 0.78); PPV, 0.62) with relatively low PPV values.

**Table 3:** Area under the ROC curves of biomarkers at baseline for prediction of overt DIC

**Biomarkers (Day 0)**	**AUC (95% CI)**	Cutoff values*	**Sensitivity**	**Specificity**	**PPV**	**NPV**
**Global markers**						
Platelet	0.65 (0.51 to 0.76)	158 (× 10^3^/μL)	0.62	0.65	0.62	0.65
PT-INR	0.61 (0.48 to 0.73)	1.3	0.62	0.63	0.61	0.64
APTT	0.61 (0.48 to 0.73)	42 (sec)	0.54	0.75	0.67	0.64
Fibrinogen	0.64 (0.51 to 0.76)	310 (mg/dL)	0.54	0.78	0.69	0.65
FDP	0.67 (0.54 to 0.78)	28 (μg/mL)	0.43	0.88	0.76	0.63
**Thrombin generation**						
TAT	0.77 (0.64 to 0.86)	15 (ng/mL)	0.67	0.85	0.81	0.72
SF	0.67 (0.54 to 0.78)	7.9 (μg/mL)	0.77	0.54	0.61	0.72
**Anticoagulant activity**						
PC	0.85 (0.76 to 0.91)	46 (%)	0.81	0.79	0.79	0.82
AT	0.76 (0.63 to 0.85)	46 (%)	0.60	0.85	0.78	0.69
**Fibrinolytic activity**						
Plasminogen	0.76 (0.63 to 0.85)	52 (%)	0.60	0.79	0.73	0.67
α2-PI	0.79 (0.67 to 0.88)	70 (%)	0.81	0.67	0.70	0.79
PAI-1	0.87 (0.78 to 0.92)	269 (ng/mL)	0.72	0.92	0.89	0.78
PIC	0.49 (0.36 to 0.63)	1.9 (μg/mL)	0.35	0.89	0.76	0.59
**Endothelial activation**						
sES	0.59 (0.45 to 0.72)	67 (ng/mL)	0.51	0.72	0.62	0.61

α2-PI, α2-plasmin inhibitor activity; APTT, activated partial thromboplastin time; AT, antithrombin activity; AUC, area under the curve; CI, confidence interval; FDP, fibrin degradation products; NPV, negative predictive value; PAI-1, plasminogen activator inhibitor-1; PC, protein C activity; PIC, plasmin-α2-plasmin inhibitor complex; PPV, positive predictive value; PT-INR, prothrombin time-international normalized ratio; ROC, receiver operating characteristic; sES, soluble E selectin; SF, soluble fibrin; TAT, thrombin- antithrombin complex. *Cutoff values were calculated to maximize the sum of sensitivity and specificity.

### Correlation and multivariate analysis to identify significant diagnostic biomarkers for subsequent development of overt DIC

To identify efficient diagnostic markers for the development of overt DIC, we undertook further analysis of significant biomarkers with AUROC >0.7 and PPV >0.7, which were superior to the results of APACHE II scores or pre-DIC scores. First, we calculated Spearman rank correlation coefficients for TAT, PC, AT, plasminogen, α_2_-PI and PAI-1 to rule out collinearity among the significant biomarkers. We found a strong and significant correlation with *r* >0.5 between each pair of PC, AT, plasminogen and α_2_-PI values (Additional file 2). However, TAT and PAI-1 were not so highly correlated with PC.

**Figure 2:**
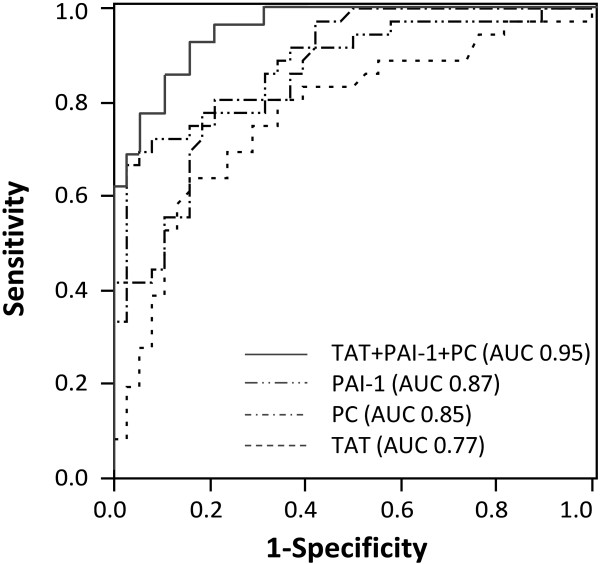
**ROC curves of TAT, PAI-1 and PC activity for prediction of overt DIC.** Area under the receiver operating characteristic curve (AUROC) for thrombin-antithrombin complex (TAT), 0.77 (95% CI, 0.64 to 0.86), plasminogen activator inhibitor-1 (PAI-1), 0.87 (0.78 to 0.92), protein C (PC), 0.85 (0.76 to 0.91), and combination of these biomarkers are described. Combination of TAT, PAI-1 and PC was superior to each marker alone (AUROC, 0.95 (vs. TAT, *P* = 0.0004; vs. PAI-1, *P* = 0.033; vs. PC, *P* = 0.025)).

Next, we conducted a multivariate stepwise logistic regression analysis, and found that TAT, PAI-1 and PC were the best combination to discriminate between patients with and without development of overt DIC. These three biomarkers remained significantly associated with overt DIC, even after adjustment for APACHE II score in separate models (TAT, *P* = 0.0002; PAI-1, *P* = 0.0001; PC, *P* <0.0001, respectively). Furthermore, the combination of TAT, PAI-1 and PC substantially improved discrimination of the development of overt DIC, compared with each marker alone (AUROC 0.95 (vs. TAT, *P* = 0.0004; vs. PAI-1, *P* = 0.033; vs. PC, *P* = 0.025), Figure 2).

### Plasma biomarkers on days 0 and 2 as predictors of 28-day mortality

**Figure 3:**
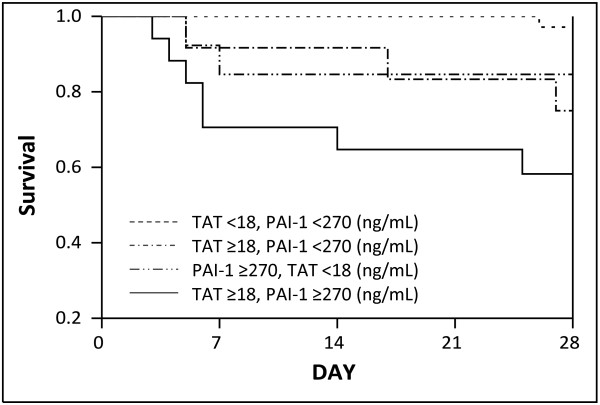
**Kaplan-Meier survival curves for patients grouped by cutoff points of TAT and PAI-1 at baseline.** The cutoff points were set at 18 ng/mL for thrombin-antithrombin complex (TAT) and 270 ng/mL for plasminogen activator inhibitor-1 (PAI-1), based on the best calculated cutoff values that maximize the sum of sensitivity and specificity for 28-day mortality.

Univariate analysis revealed that only TAT and PAI-1 at baseline were significant predictors of 28-day mortality among the biomarkers that had good discriminative power for the development of overt DIC (Table 4 and Additional file 3). Based on the best calculated cutoff values, cutoff points at baseline were set at 18 ng/mL for TAT and 270 ng/mL for PAI-1. The Kaplan-Meier survival curve for patients with sepsis demonstrated that TAT >18 ng/mL and/or PAI-1 >270 ng/mL on admission were significantly correlated with higher mortality (*P* = 0.0024, Figure 3).

**Table 4:** Area under ROC curves of Day 0 and Day 2 biomarkers for prediction of mortality

**Biomarkers**	**ICU day**	**AUC (95% CI)**	Cutoff values*	**Sensitivity**	**Specificity**	**PPV**	**NPV**
**Global markers**							
Platelet	Day 0	0.58 (0.41 to 0.74)	117 ( × 10^3^/μL)	0.41	0.79	0.32	0.84
	2	0.81 (0.64 to 0.91)	66 ( × 10^3^/μL)	0.81	0.79	0.48	0.94
PT-INR	Day 0	0.53 (0.34 to 0.72)	1.2	0.53	0.71	0.31	0.86
	2	0.68 (0.47 to 0.84)	1.5	0.61	0.81	0.43	0.89
FDP	Day 0	0.61 (0.42 to 0.76)	21 (μg/mL)	0.61	0.65	0.29	0.87
	2	0.61 (0.41 to 0.77)	22 (μg/mL)	0.67	0.65	0.31	0.89
**Thrombin generation**							
TAT	Day 0	0.77 (0.62 to 0.87)	18 (ng/mL)	0.81	0.77	0.46	0.94
	2	0.83 (0.65 to 0.93)	16 (ng/mL)	0.67	0.92	0.67	0.92
**Anticoagulant activity**							
PC	Day 0	0.64 (0.45 to 0.79)	37 (%)	0.53	0.75	0.35	0.87
	2	0.76 (0.53 to 0.89)	22 (%)	0.61	0.97	0.82	0.91
**Fibrinolytic activity**							
Plasminogen	Day 0	0.64 (0.45 to 0.79)	61 (%)	0.81	0.52	0.29	0.91
	2	0.75 (0.57 to 0.87)	50 (%)	0.81	0.67	0.38	0.93
PAI-1	Day 0	0.81 (0.64 to 0.91)	269 (ng/mL)	0.85	0.71	0.38	0.96
	2	0.91 (0.79 to 0.96)	81.4 (ng/mL)	0.69	0.97	0.82	0.94

AUC, area under the curve; CI, confidence interval; FDP, fibrin degradation products; NPV, negative predictive value; PAI-1, plasminogen activator inhibitor-1; PC, protein C activity; PPV, positive predictive value; PT-INR, prothrombin time-international normalized ratio; ROC, receiver operating characteristic; TAT, thrombin-antithrombin complex.

*Cutoff values were calculated to maximize the sum of sensitivity and specificity.

Most of the studied Day 2 markers had higher AUROCs for prediction of 28-day mortality compared with Day 0 markers (Table 4 and Additional file 3). Among the Day 2 biomarkers, TAT, SF and PAI-1 remained statistically significant for prediction of 28-day mortality after adjustment for APACHE II score (*P* = 0.0016, *P* <0.0001, *P* <0.0001, respectively).

## Discussion

The main findings of our study were as follows. 1) Coagulopathy developed in the initial phase of sepsis, and the severity of hemostatic biomarker abnormalities on the day of admission was associated with the subsequent development of overt DIC. 2) Among all the studied biomarkers, TAT, PAI-1 and PC had the best discriminative power for the patients who newly developed overt DIC. 3) However, only TAT and PAI-1 on Day 0 were significant predictors of 28-day mortality among the diagnostic biomarkers for the development of overt DIC. In contrast, Day 2 markers had higher predictive power for 28-day mortality compared with Day 0 markers, suggesting that persistence of severe coagulopathy was correlated with mortality.

Inflammation and coagulation constitute two host defense systems with complementary roles against infection [13], which means that an overwhelming systemic inflammatory reaction in sepsis is accompanied by severe coagulopathy, and both may contribute to tissue damage in the early phase of sepsis. In our study, most patients with sepsis exhibited coagulation and fibrinolytic abnormalities at the time of ICU admission, which is consistent with the data from the PROWESS trial [1]. In addition, most hemostatic biomarkers measured on ICU admission were associated with subsequent fulfillment of overt DIC criteria. These results support the hypothesis that coagulopathy is present in the initial phase of sepsis, and the strategy to identify markers of acute ongoing coagulopathy, rather than to detect pre-DIC state, may be necessary for the early diagnosis of septic DIC.

The pathogenesis of DIC is primarily due to excess production of thrombin [20]. In sepsis, anticoagulation impairment and insufficient fibrinolysis also contribute to thrombin generation and fibrin deposition. Anticoagulation pathways such as the antithrombin and protein C systems are impaired because of increased consumption, decreased protein synthesis, extravasation and degradation by several proteolytic enzymes such as neutrophil elastase [21,22]. The fibrinolytic system is largely suppressed by increased production of PAI-1, which is a principal inhibitor of this system [23,24]. In our study, increased levels of TAT and PAI-1, and decreased PC activity, were observed at the time of ICU admission and each independently discriminated the patients who developed overt DIC from those who did not. Our findings indicate that activation of coagulation, anticoagulation impairment and insufficient fibrinolysis develop early in the course of sepsis, and these three mechanisms should be evaluated individually for the diagnosis of DIC in patients with sepsis.

In this study, we found that TAT, a marker of thrombin generation, and PAI-1, which is induced by pro-inflammatory cytokines, were highest at baseline and improved when diagnosis of DIC was made in patients who developed overt DIC. These significant trends were obvious in survivors with overt DIC. In non-survivors with overt DIC, elevated levels of TAT and PAI-1 persisted during the study period. Similar trends in those biomarkers were observed in an experimental model of sepsis and in clinical studies [25,26]. TAT and PAI-1 have short half-lives and they are produced early in the course of septic coagulopathy, while other biomarkers, such as platelets, PT-INR or PC, are the markers of consumption. The differences in those biomarkers over time between survivors and non-survivors indicate that TAT and PAI-1 may well reflect disease progress in septic coagulopathy.

Current criteria for early diagnosis of DIC have some potential limitations. Considering easy implementation, most criteria, including ISTH non-overt DIC and JAAM acute DIC criteria, use readily available coagulation tests for scoring. However, it is clear that global coagulation tests, such as PT and platelet count, primarily reflect the result of consumption and impaired synthesis rather than direct ongoing coagulopathy. Kinasewitz *et al*. [27] and Dhainaut *et al*. [28] established a simple diagnostic scoring system for the acute phase of septic coagulopathy, but these systems depend partly on worsening trends of global markers, which take at least two days to identify.

Several hemostatic molecular biomarkers, including AT, PC, TAT, PIC and PAI-1, have also been evaluated in patients with sepsis, but the reported results were inconsistent [1,24,25,28-30]. Several possible explanations could account for these conflicting results. First, we demonstrated dynamic changes in the biomarkers within a few days in the initial phase of sepsis, which is consistent with previous studies [1,25]; therefore, the timing of biomarker measurement is important for interpretation of the results. Second, the cutoff value is another factor that influences the diagnostic ability of biomarkers. Oh *et al*. [6] and Egi *et al*. [31] evaluated the cutoff value of the lower limit of normal (70%) in AT levels for ISTH non-overt DIC criteria, and showed that the diagnostic ability for overt DIC did not improve by adding AT to non-overt DIC criteria. In our study, AT activity, as well as PC, was decreased below the lower limit of normal, even in most of the patients without overt DIC, and the cutoff value of AT level to discriminate patients with and without overt DIC was much lower (46.1%). Last, most of the previous studies evaluated the impact of hemostatic biomarkers on prognosis in patients with sepsis. We found that some plasma biomarkers at baseline were good predictors for the development of overt DIC, but were less predictive for 28-day mortality compared with Day 2 markers, which indicates that persistence of coagulopathy, rather than just the development of it, influences the prognosis in patients with sepsis. In addition, multiple interactive systemic factors other than coagulopathy would be involved in the pathogenesis of organ failure and the risk of mortality. We, therefore, evaluated diagnostic and prognostic values of biomarkers individually.

There were some potential limitations to our study. First, this was a prospective observational study conducted in a single center with a relatively small population size. Although the overall rate of DIC matched that in previous studies [3,32], our cohort included fewer pneumonia patients, who often die from respiratory failure rather than multiple organ failure, including DIC. A large validation study is needed to confirm our results. Second, there is no gold standard for diagnosis of or the criteria for intervention in sepsis-associated coagulopathy. We used the ISTH overt DIC criteria as the diagnostic standard, considering coagulopathy that fulfilled these criteria would be severe enough to be eligible for intervention. Third, although our management of sepsis followed the SSCG guidelines, and did not deviate from standard care, prophylactic anticoagulation and interventions, such as blood transfusion as well as AT substitution, may have influenced the levels of hemostatic biomarkers except for baseline profile, and their relationship with the scores of overt DIC. Last, our study lacked explanations about why TAT, PAI-1 and PC were the best diagnostic markers for overt DIC. We found a strong correlation among AT, PC, plasminogen and α_2_-PI at baseline. Considering that the same mechanism of consumption might be the main reason for decreased activity of those biomarkers [33], it is unclear why PC had superior diagnostic ability. Of particular interest is the contrast between the diagnostic value of TAT and another thrombin generation marker, SF. One possible explanation is the differences in half-life or mechanisms of clearance, where TAT has a shorter half-life (10 to 15 minutes), compared with SF (several hours). Further study is needed to better understand the processes of these biomarkers, and for the development of new therapeutic strategies in septic DIC.

## Conclusions

The results of our study provide evidence that almost half of the patients developed severe coagulopathy in the initial phase of sepsis, which was demonstrated by baseline abnormalities in hemostatic biomarkers and their strong association with subsequent fulfillment of overt DIC criteria. In particular, a single determination of TAT, PAI-1 and PC activity at ICU admission allowed early identification of severe coagulopathy, or DIC, leading to early intervention for patients with sepsis.

## Key messages

•The present study showed that coagulopathy was frequently observed in the initial phase of sepsis, and severe coagulation and fibrinolytic abnormalities were strongly associated with subsequent development of overt DIC.

•Among the 14 plasma biomarkers evaluated, TAT, PAI-1 and PC activity on ICU admission were the best combination to discriminate between patients with and without overt DIC.

•In terms of predicting mortality, only TAT and PAI-1 were significant predictors of 28-day mortality at the time of ICU admission.

## Abbreviations

α2-PI: α_2_-plasmin inhibitor; APACHE: Acute Physiology and Chronic Health Evaluation; AT: Antithrombin; AUROC: Area under the receiver operating curve; DIC: Disseminated intravascular coagulation; FDP: Fibrin degradation product; ISTH: International Society on Thrombosis and Haemostasis; JAAM: Japanese Association for Acute Medicine; NPV: Negative predictive value; PAI-1: Plasminogen activator inhibitor-1; PC: Protein C; PIC: Plasmin-α_2_-plasmin inhibitor complex; PPV: Positive predictive value; PT-INR: Prothrombin time-international normalized ratio; ROC: Receiver operating characteristic; sES: soluble E-selectin; SF: Soluble fibrin; SOFA: Sequential Organ Failure Assessment; TAT: Thrombin-antithrombin complex.

## Competing interests

The authors declare that they have no competing interests.

## Authors’ contributions

KK conceived and designed the study. KK and SN prepared the data for analysis. KK conducted the data analysis. SM assisted with interpretation of the results. YS, JM and SN supervised the study. KK and SM drafted the article. All authors read and approved the manuscript. KK and SM take responsibility for the paper as a whole.

## Supplementary Material

Additional file 1: Figure S1 Time course of biomarkers from baseline to Day 3. Fibrinogen, soluble fibrin (SF), plasminogen, α_2_-plasmin inhibitor (α2-PI), plasmin-α_2_-plasmin inhibitor complex (PIC) and soluble E-selectin (sES) for patients with and without subsequent development of overt disseminated intravascular coagulation (DIC) (gray vs. white bars), and for survivors (dotted line) and non-survivors (solid line) among patients with overt DIC. Data are expressed as mean and 95% CI. *P <0.05 between patients with and without overt DIC on the same day. **P <0.05 between patients on Day 0 versus Day 2. ^+^*P* <0.05 between survivors and non-survivors with overt DIC on the same day. Click here for file

Additional file 2: Figure S2 Correlation of plasma biomarkers at baseline with each other. The correlation graphs and Spearman rank correlation coefficients (*r* value) are shown here. Click here for file

Additional file 3: Table S1 Area under ROC curves of Day 0 and Day 2 biomarkers for prediction of mortality. Click here for file
