# Supplementary figures and images for: Combination of thrombin-antithrombin complex, plasminogen activator inhibitor-1, and protein C activity for early identification of severe coagulopathy in initial phase of sepsis: a prospective observational study
Source: Crit Care. 2014 Jan 13;18(1):R13. doi: 10.1186/cc13190 (PMC4056264; doi:10.1186/cc13190)

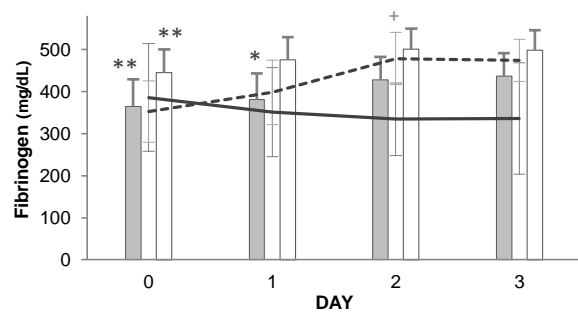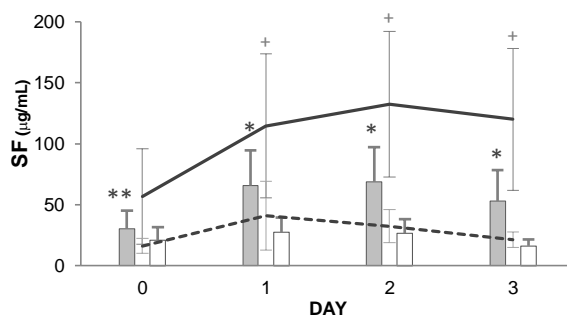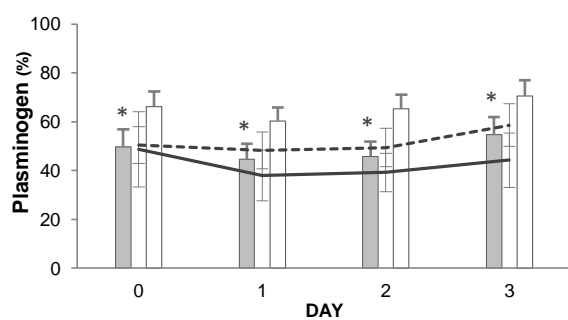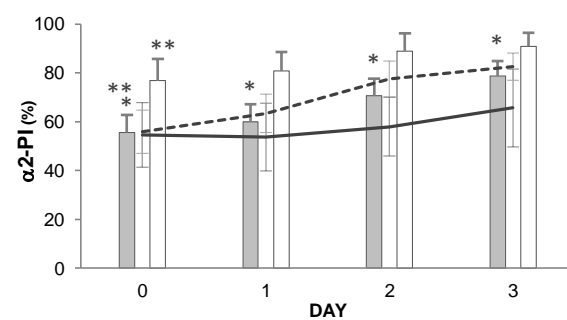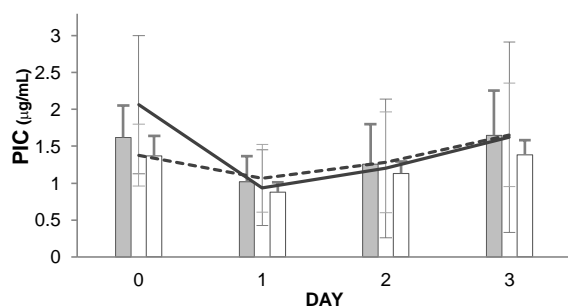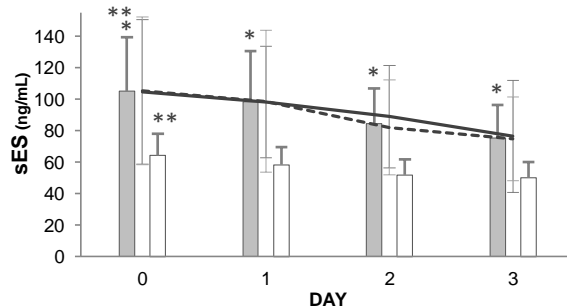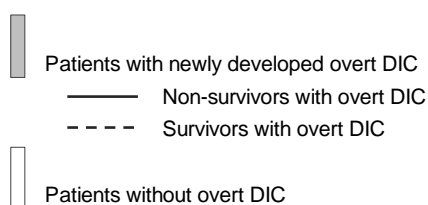

Supplement: Additional file 1: Figure S1 — Time course of biomarkers from baseline to Day 3. Fibrinogen, soluble fibrin (SF), plasminogen, α2-plasmin inhibitor (α2-PI), plasmin-α2-plasmin inhibitor complex (PIC) and soluble E-selectin (sES) for patients with and without subsequent development of overt disseminated intravascular coagulation (DIC) (gray vs. white bars), and for survivors (dotted line) and non-survivors (solid line) among patients with overt DIC. Data are expressed as mean and 95% CI. *P <0.05 between patients with and without overt DIC on the same day. **P <0.05 between patients on Day 0 versus Day 2. +P <0.05 between survivors and non-survivors with overt DIC on the same day. [file cc13190-S1.pdf]

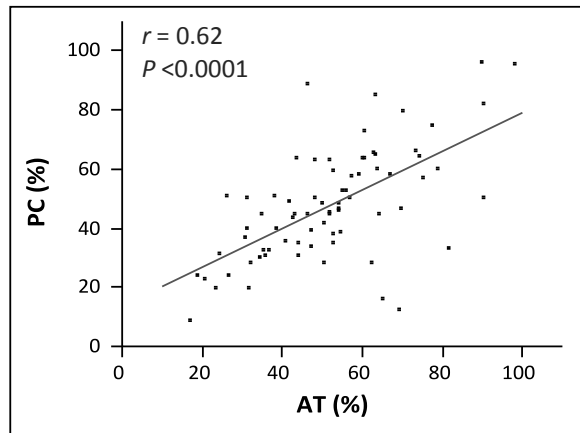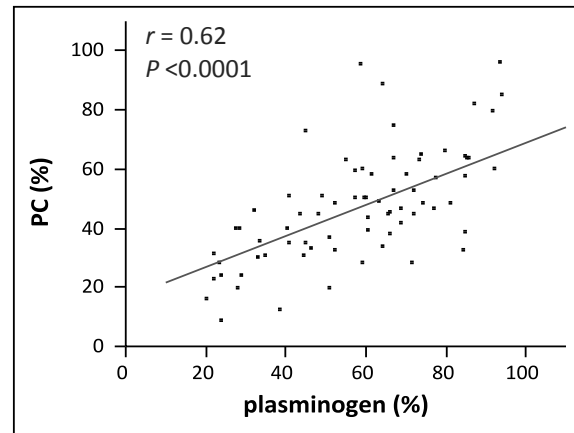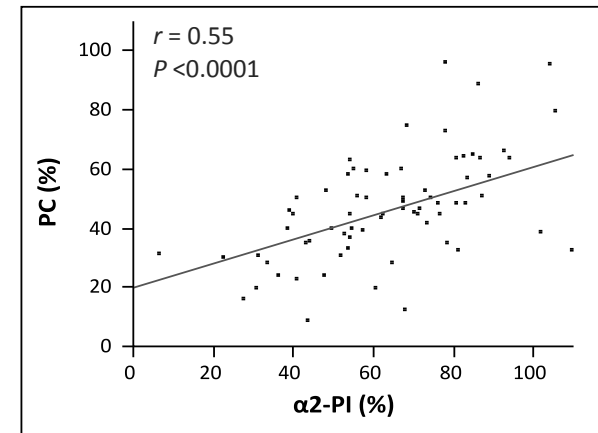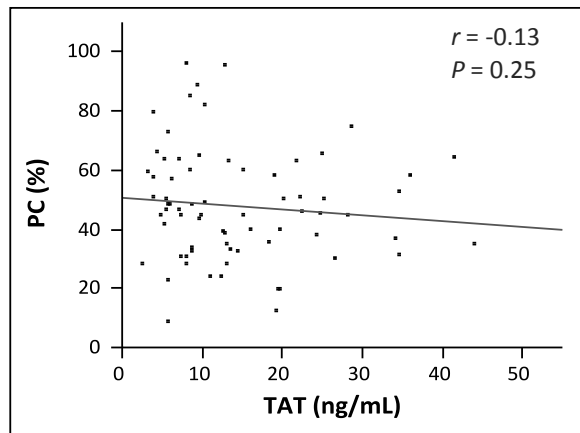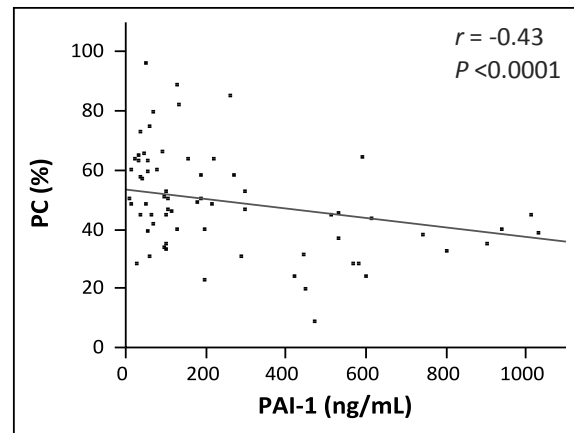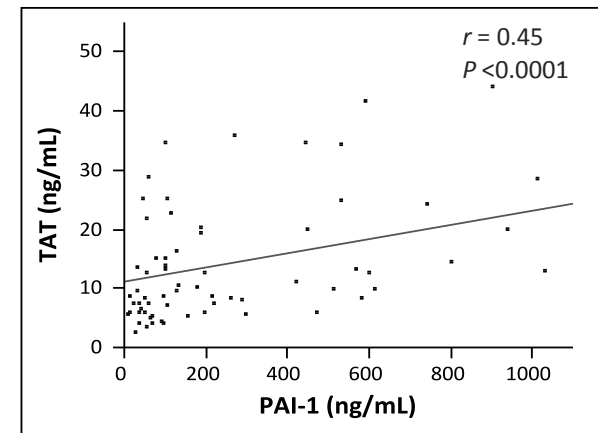

Supplement: Additional file 2: Figure S2 — Correlation of plasma biomarkers at baseline with each other. The correlation graphs and Spearman rank correlation coefficients (r value) are shown here. [file cc13190-S2.pdf]
