# Supplementary material for: Combination of thrombin-antithrombin complex, plasminogen activator inhibitor-1, and protein C activity for early identification of severe coagulopathy in initial phase of sepsis: a prospective observational study
Source: Crit Care. 2014 Jan 13;18(1):R13. doi: 10.1186/cc13190 (PMC4056264; doi:10.1186/cc13190)
Supplement: Additional file 3: Table S1 — Area under ROC curves of Day 0 and Day 2 biomarkers for prediction of mortality. [file cc13190-S3.docx]

| **Table S1 Area under ROC curves of Day-0 and Day-2 biomarkers for prediction of mortality** | | | | | | | | |
| --- | --- | --- | --- | --- | --- | --- | --- | --- |
| **Biomarkers** | **ICU day** | | **AUC (95%CI)** | **Cutoff values*** | **Sensitivity** | **Specificity** | **PPV** | **NPV** |
| **Global markers** |  |  |  |  |  |  |  |  |
| APTT | Day 0 |  | 0.49 (0.32 to 0.67) | 84 (sec) | 0.21 | 0.95 | 0.51 | 0.83 |
|  | 2 |  | 0.81 (0.59 to 0.92) | 68 (sec) | 0.73 | 0.89 | 0.61 | 0.93 |
|  |  |  |  |  |  |  |  |  |
| Fibrinogen | Day 0 |  | 0.49 (0.31 to 0.66) | 692 (mg/dL) | 0.21 | 0.95 | 0.51 | 0.83 |
|  | 2 |  | 0.76 (0.56 to 0.89) | 435 (mg/dL) | 0.87 | 0.65 | 0.37 | 0.95 |
| **Thrombin generation** |  |  |  |  |  |  |  |  |
| SF | Day 0 |  | 0.72 (0.52 to 0.85) | 13 (μg/mL) | 0.77 | 0.64 | 0.31 | 0.93 |
|  | 2 |  | 0.89 (0.73 to 0.96) | 30 (μg/mL) | 0.92 | 0.74 | 0.44 | 0.98 |
| **Anticoagulant activity** |  |  |  |  |  |  |  |  |
| AT | Day 0 |  | 0.59 (0.38 to 0.77) | 41 (%) | 0.61 | 0.81 | 0.43 | 0.89 |
|  | 2 |  | 0.67 (0.45 to 0.84) | 36 (%) | 0.51 | 0.93 | 0.61 | 0.91 |
| **Fibrinolytic activity** |  |  |  |  |  |  |  |  |
| α2-PI | Day 0 |  | 0.59 (0.41 to 0.75) | 61 (%) | 0.67 | 0.64 | 0.31 | 0.87 |
|  | 2 |  | 0.77 (0.55 to 0.90) | 64 (%) | 0.67 | 0.87 | 0.56 | 0.91 |
|  |  |  |  |  |  |  |  |  |
| PIC | Day 0 |  | 0.58 (0.37 to 0.77) | 1.8 (μg/mL) | 0.53 | 0.82 | 0.42 | 0.88 |
|  | 2 |  | 0.39 (0.21 to 0.61) | 2.8 (μg/mL) | 0.21 | 0.96 | 0.61 | 0.83 |
| **Endothelial activation** |  |  |  |  |  |  |  |  |
| sES | Day 0 |  | 0.63 (0.42 to 0.80) | 90 (ng/mL) | 0.54 | 0.79 | 0.35 | 0.89 |
|  | 2 |  | 0.64 (0.43 to 0.82) | 90 (ng/mL) | 0.55 | 0.83 | 0.41 | 0.89 |
| α2-PI, α2-plasmin inhibitor activity; APTT, activated partial thromboplastin time; AT, antithrombin activity; AUC, area under the curve; CI, confidence interval; NPV, negative predictive value; PIC, plasmin-α2-plasmin inhibitor complex; PPV, positive predictive value; ROC, receiver operating characteristic; sES, soluble E selectin; SF, soluble fibrin. *Cutoff values were calculated to maximize the sum of sensitivity and specificity. | | | | | | | | |
